# Preclinical Studies of the Biosafety and Efficacy of Human Bone Marrow Mesenchymal Stem Cells Pre-Seeded into β-TCP Scaffolds after Transplantation

**DOI:** 10.3390/ma11081349

**Published:** 2018-08-03

**Authors:** Mar Gonzálvez-García, Carlos M. Martinez, Victor Villanueva, Ana García-Hernández, Miguel Blanquer, Luis Meseguer-Olmo, Ricardo E. Oñate Sánchez, José M. Moraleda, Francisco Javier Rodríguez-Lozano

**Affiliations:** 1 Cell Therapy Unit, IMIB-University Hospital Virgen de la Arrixaca, Faculty of Medicin, University of Murcia, 30120 Murcia, Spain; margonzalvez@yahoo.com (M.G.-G.); amgh8@hotmail.com (A.G.-H.); miguelblanquer@gmail.com (M.B.); l.meseguer.doc@gmail.com (L.M.-O.); jmoraled@um.es (J.M.M.); 2 Service of Oral and Maxillofacial Surgery, Clinical University Hospital Virgen de la Arrixaca, 30120 Murcia, Spain; villanuevasan@gmail.com; 3 Inflammation and Experimental Surgery Unit, CIBERehd, Institute for Bio-Health Research of Murcia, Clinical University Hospital Virgen de la Arrixaca, 30120 Murcia, Spain; cmmarti@um.es; 4 School of Dentistry, University of Murcia, 30003 Murcia, Spain; reosan@um.es; 5 Unidad de Pacientes Especiales y Gerodontología, Universidad de Murcia, IMIB-Arrixaca, Hospital Morales Meseguer, 30008 Murcia, Spain

**Keywords:** preclinical biosafety, bone substitute, mesenchymal stem cells, β-tricalcium phosphate, tissue engineering

## Abstract

*Background*: Cell-Based Therapies (CBT) constitute a valid procedure for increasing the quantity and quality of bone in areas with an inadequate bone volume. However, safety and efficacy should be investigated prior to clinical application. The objective of this study was to evaluate the biodistribution, safety and osteogenic capacity of bone marrow-derived human mesenchymal stem cells (*h*BMMSCs) pre-seeded into β-tricalcium phosphate (TCP) and implanted into NOD/SCID mice at subcutaneous and intramuscular sites. *Methods*: *h*BMMSCs were isolated, characterized and then cultured in vitro on a porous β-TCP scaffold. Cell viability and attachment were analyzed and then *h*BMMSCs seeded constructs were surgically placed at subcutaneous and intramuscular dorsal sites into NOD/SCID mice. Acute and subchronic toxicity, cell biodistribution and efficacy were investigated. *Results*: There were no deaths or adverse events in treated mice during the 48-hour observation period, and no toxic response was observed in mice. In the 12-week subchronic toxicity study, no mortalities, abnormal behavioral symptoms or clinical signs were observed in the saline control mice or the *h*BMMSCs/β-TCP groups. Finally, our results showed the bone-forming capacity of *h*BMMSCs/β-TCP since immunohistochemical expression of human osteocalcin was detected from week 7. *Conclusions*: These results show that transplantation of *h*BMMSCs/β-TCP in NOD/SCID mice are safe and effective, and might be applied to human bone diseases in future clinical trials.

## 1. Introduction

Bone marrow is a source of mesenchymal stromal stem cells (MSCs) which have demonstrated in vivo and in vitro ability to differentiate into osteoblasts and chondrocytes, thus providing tissue repair capacities [1,2]. Their functional properties have been confirmed in several studies using autologous human bone marrow mesenchymal stem cells (*h*BMMSC) for bone repairing and tissue healing [3]. *h*BMMSC represent a cell type with a high potential for bone regeneration [4] as a result of their multipotential differentiation capacity, including differentiation into the osteogenic lineage, which constitutes a very valuable tool in medicine, specifically for tissue engineering in traumatology or maxillofacial applications [5,6]. When hBMMSCs are seeded into a scaffold, the final product brings together the osteoinductive and osteoconductive properties of the biomaterial and the regenerative and homeostatic properties of the cells. Therefore, this approach can provide an alternative to autogenous bone grafting that usually adds morbidity to the patients [7].

Cell-therapy approaches constitute one of the most promising instruments to enhance the reconstruction of both hard and soft tissues [8,9]. Nevertheless, cell dose and viability are always a problem when we move from the bench to preclinical, or even further, to the clinical setting. Therefore, this point remains to be optimized [10].

Cell-Based Therapies (CBT) are a promising approach to a wide variety of medical conditions that currently do not have satisfactory treatments. However, differentiation and proliferation potential of CBT involve new safety concerns that are not considered for conventional drug products [10]. Preclinical studies are needed to address the safety and efficacy of an investigational stem cell-based product before to move to the clinic. The development of new 3D scaffolds using advanced strategies [11,12], the mechanism of action of the mesenchymal stem cells and the most efficient route of administration have to be investigated in animal models that ideally should replicate human disease without compromising the ability of human cells to engraft and survive [13]. One step higher, MSCs from different sources are currently being tested as investigational medicinal products in several clinical trials (clinicaltrials.gov) [13]. However, many clinical trials have failed to demonstrate efficacy results because, as we have previously mentioned, critical aspects such as cell dose, homing, engraftment, and biodistribution in vivo of these “living drugs” are difficult to extrapolate from preclinical models [14]. In Europe, MSCs are somatic cell-therapy products, referred to as advanced therapy medicinal products (ATMPs) and are subject to European Regulation No. 1394/2007 [15].

The aim of this study was to test the biodistribution and security profile of *h*BMMSCs pre-seeded into β-tricalcium phosphate (TCP) after subcutaneous/intramuscular transplantation. In addition, the safety in terms of toxicity of the procedure and its capacity of osteocalcin production was evaluated.

## 2. Material and Methods

### 2.1. Isolation and Culture of Bone Marrow-Derived hBMMSCs

Multipotent *h*MSCs were isolated from bone marrow as described previously [16]. The study was approved by the Institutional Ethics Committee (Virgen de la Arrixaca University Hospital ID: 101212/1/AEMPS), while all patients signed an informed consent. For isolation, the aspirated material was transferred into transfer bags containing heparin. The mononuclear cell fraction was obtained using Ficoll density gradient media and a cell washing closed automated SEPAX™ System (Biosafe, Eysines, Switzerland). After estimating the viability with trypan blue staining, cells were plated out in 75 cm^2^ culture flasks (Sarstedt, Nümbrecht, Germany) with 10 mL of basal culture growth medium (GM). The GM used was α-MEM (Minimum Essential Media) medium (Invitrogen, Carlsbad, CA, USA), supplemented with 15% fetal bovine serum (FBS, Invitrogen), 100 mM L-ascorbic acid phosphate (Sigma-Aldrich, Steinheim, Germany) and antibiotics/antimycotics before incubating at 37 °C in 5% CO_2_. Cells in passage 3 were used for both in vitro and in vivo experiments.

### 2.2. Immunophenotypic Profiles of hBMMSC Cultures

*h*BMMSCs were analyzed by flow cytometry for mesenchymal (CD90, CD73), endothelial (CD105/endoglin,), hematopoietic (CD34, CD45) and HLA-DR stem cell (SC) markers, as previously described [17,18,19]. Single cell suspensions obtained by culture trypsinization were labelled or surface markers with fluorochrome-conjugated antibodies: CD73-PE, CD90-APC, CD105-FITC, HLA-DR-FITC, CD34-APC and CD45-FITC (Human MSC Phenotyping Cocktail, Miltenyi Biotec, Bergisch Gladbach, Germany).

### 2.3. Human Bone Marrow-Derived Mesenchymal Stem Cells (hBMMSCs) Seeded into Scaffold (hBMMSCs/ β-TCP) Constructs Preparation

Synthetic β-Tricalcium phosphate (Cellplex^TM^ TCP, Wright Medical Technology, Inc., Arlington, TN, USA) with size of 0.7–1.4 mm, a porosity of 60%, and a pore size of 100–400 pm was used as carrier. This dimension was appropriate for the specific application in the subcutaneous/intramuscular implantation. Prior to cell seeding, sterile β-TCP granules were pre-wetted for 1 h in complete medium. For cell seeding in the study group, *h*BMMSCs were trypsinized, centrifuged and resuspended in an appropriate volume; after cell counting, the density of cells in suspension was adjusted to about 1 × 10^6^ cells. For the control group, β-TCP granules were pre-wetted with complete culture medium free of cells.

### 2.4. Cell Viability Assay

For this purpose, the MTT [3-(4,5-dimethylthiazol-2-yl)-2,5-diphenyltetrazolium bromide] assay was used, as previously described [20]. The cells/scaffold constructs were initially loaded with 1.0 × 10^4^ cells/well in 96-well plates. After 1, 7 and 14 days, MTT (0.5 mg/mL in GM) was added to each cell/scaffold construct. Cells were seeded on β-TCP, as described above and 3 to 5 granules, depending on the granule size, and incubated for 4 h at 37 °C and 5% CO_2_. The MTT insoluble formazan was then dissolved by means of DMSO (Dimethyl sulfoxide) that was applied for 2–4 h to the constructs at 37 °C. The optical density (OD) was measured against blank (DMSO) at a wavelength of 570 nm and a reference filter of 690 nm by an automatic microplate reader (ELx800; Bio-Tek Instruments, Winooski, VT, USA). Cell-free scaffolds incubated under the same conditions were used as reference controls and their OD values were subtracted from those obtained from the corresponding *h*BMMSCs /scaffold constructs. Population doubling number (PDN) was then calculated for the cells from days 1 to 14 using the cell number at day 1 as the seeding cell number (N0) and the day 14 as the harvested cell number (N1). The PDN was calculated with the following equation: Log10 (N1/N0) × 3.33 [21].

### 2.5. Scanning Electron Microscopy (SEM) Study of hBMMSCs Seeded on β-TCP

To evaluate the cell attachment of *h*BMMSCs adhered to β-TCP, study periods of 24 h, and 7 and 15 days were established. Then, *h*BMMSCs were directly seeded onto β-TCP granules at a density of 5 × 10^4^ cells/mL. After 24 h, 7 and 15 days of culture, the samples seeded with *h*BMMSCs were primarily fixed in a solution of 3% glutaraldehyde, 0.1 M Sucrose, 0.1 M sodium cacodylate for 45 min at 4 °C. Then, they were rinsed again and dehydrated increasing concentrations (50–100% *v*/*v*) of ethanol and hexamethyldisilizane. The samples were dried in a critical point drier CPDO2 (Balzers Union, Liechtenstein, Germany) sputter-coated with a 20 nm thick layer of gold-palladium and observed under a SEM (JSM-6390 LV, JEOL, Tokyo, Japan).

### 2.6. In vivo hBMMSCs/β-TCP Constructs Transplantation

Thirty female NOD/SCID mice (Charles River Laboratories, Inc., Wilmington, MA, USA) with an average age of 6 weeks were used in this study. All animal experiments were conducted in accordance with the European Union guidelines for experimental animal use. The study protocol was approved by the Ethical Committee for Animal Care of the University of Murcia, Murcia, Spain (101212/1/AEMPS).

Mice were anesthetized intraperitoneally with a solution of ketamine (Renaudin, Aïnhoa, France, 100 mg/kg) and xylazine (Rompun, Bayer AG, Leverkusen, Germany, 10 mg/kg) and fixed on the board. After an aseptic preparation was applied to the skin. A subcutaneous incision was made at the middle of the dorsum.

The mice were randomly divided into two groups:

Group 1 formed by 25 NOD/SCID mice. A subcutaneous pocket was bluntly created in the left paravertebral area. 5 granules of *h*BMMSCs/β-TCP constructs were transplanted into the pockets, and the wound was suture closed. In addition, 5 granules of *h*BMMSCs/β-TCP construct was transplanted intramuscularly in the right paravertebral area.

Group 2 formed by 5 NOD/SCID mice. A subcutaneous pocket was bluntly created in the left paravertebral area. 5 constructs (1%PBS/β-TCP) were transplanted into the pockets, and the wound was suture closed. Also, 5 constructs (1%PBS/β-TCP) were transplanted intramuscularly in right paravertebral area.

Food and water was given *ad libitum* and the individuals’ normal values for complications, abnormal locomotor activity, food and water consumption were recorded at different time points: 1 day, 2 days, 1 week, 2 weeks, 5 weeks, 7 weeks, 9 weeks and 12 weeks; 14 organs (lung, heart, kidney, spleen, tibialis anterior muscle, brain, inguinal fat pad, bone marrow, stomach, intestine, liver, ovary, blood, knee joint) were harvested and frozen at −80 °C.

### 2.7. Acute and Subchronic Toxicity Study

To assess the acute toxicity, the animals from both groups were observed continuously before surgery, at each hour for the first 4 hours and then at 6 hours interval for the next 48 hours after construct transplantation, to observe any deaths or abnormal locomotor activities. All mice were scored using a traditional welfare scoring system [22]. Values between 0–4 are considered a good welfare status, values of 5–9 indicate some kind of suffering, while 10–14 suggests that the mouse is in a state of considerable suffering. Finally, a score of between 15 and 19 (vocalization, self-mutilation, restlessness/stillness) is associated with intense pain and the animal should be sacrificed immediately. In addition, acute organ toxicity was evaluated by histological analysis 24 h and 48 h after surgery.

Subchronic toxicity was evaluated 1, 2, 5, 7, 9 and 12 weeks after surgery in all groups. The body weights and welfare status were recorded weekly. During the entire course of the study, animals were observed daily. In addition, subchronic organ toxicity was evaluated by histological analysis at the same time points.

### 2.8. Biodistribution

*h*BMMSCs were detected in mouse tissues using the quantitative polymerase chain reaction (qPCR) technique described by François et al. [23]. Genomic DNA from fresh tissues was prepared using the QIAamp DNA Mini Kit from Qiagen according to the manufacturer’s instructions. The amount of human DNA in each sample was quantified by amplification of the human beta-globin gene, while endogenous mouse RAPSYN gene (Receptor-Associated Protein at the Synapse), served as internal control. Absolute standard curves were generated for the human beta-globin and mouse RAPSYN genes. One hundred nanograms of purified DNA from several tissues was amplified using Taqman Fast Advanced Master Mix and a Step-One Plus Real Time PCR (Polymerase Chain Reaction) system (Applied Biosytems, Foster City, CA, USA). The primers and probe for human beta-globin were: forward primer 5′GTGCACCTGACTCCTGAGGAGA3′ and reverse primer 5′CCTTGATACCAACCTGCCCAGG3′; the probe labelled with fluorescent reporter and quencher was 5′FAM-AAGGTGAACGTGGATGAAGTTGGTGG-TAMRA-3′. The primers and probe for mouse RAPSYN gene were forward primer 5′ACCCACCCATCCTGCAAAT3′ and reverse primer 5′ACCTGTCCGTGCTGCAGAA3′; the probe labelled with fluorescent reporter and quencher was 5′FAM-CGGTGCCAGTGATGAGGTTGGTC-TAMRA-3′. Likewise, human DNA was isolated from hMSC culture and used as a positive control [24].

### 2.9. Anatomic Pathology Examination

Representative samples from constructs and brain, lung, heart, liver, kidney, gut, spleen, lymph node, bone marrow were fixed in 4% buffered formalin (Panreac Quimica, Barcelona, Spain) for 48 h. Constructs and bone marrow were additionally decalcified in a formic-acid-based commercial solution (TBD-2, Thermo, Madrid, Spain) for 12–16 h. Samples were then washed, processed and paraffin-embedded. Sections were obtained and stained with hematoxylin and eosin (H&E) for standard histological analyses. To study the presence of human osteocalcin producer cells, a standard indirect ABC immunohistochemical staining was performed, using a specific polyclonal rabbit human anti-osteocalcin antibody (LsBio, Seattle, WA, USA) with a commercial kit EnVision FlexTM, (Dako, Carpinteria, CA, USA)). All samples were evaluated with a conventional light microscope (Axio Scope AX10, Zeiss, Oberkochen, Germany), with attached digital camera (Axio Cam Icc3, Carl Zeiss, Jenna, Germany).

### 2.10. Statistics

Data were analyzed using the SPSS software (version 19, SPSS, Inc., Chicago, IL, USA). Statistical analysis was conducted using the Mann-Whitney U-test or Student’s t-test (others). *p* < 0.05 was interpreted as denoting statistical significance.

## 3. Results

### 3.1. Characterization of hBMMSCs In Vitro Experiments

**Figure 1:**
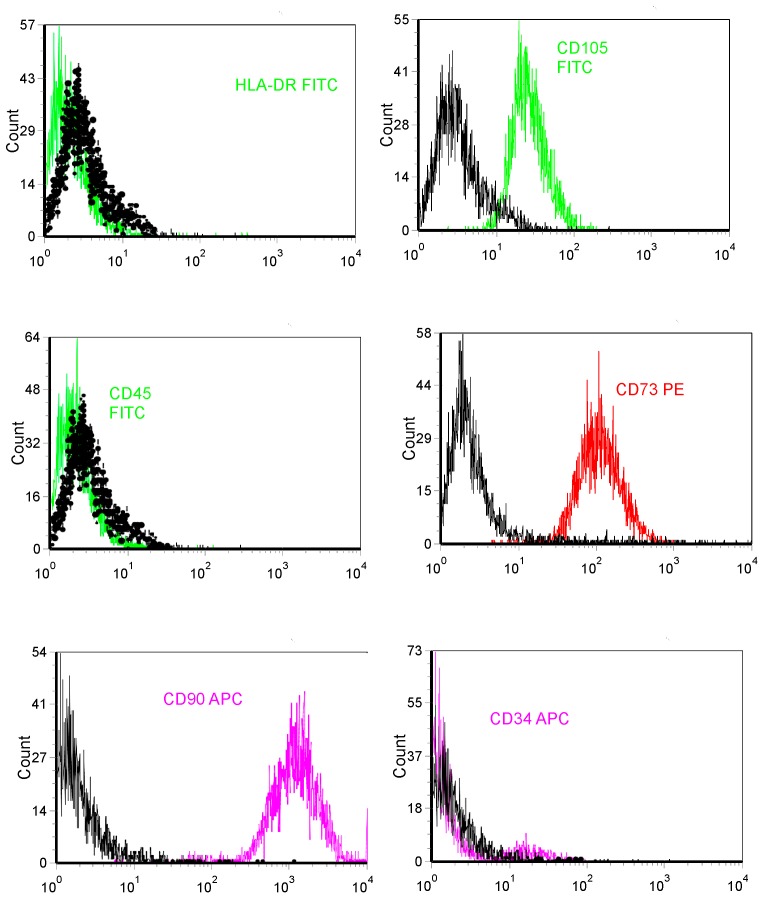
Immunophenotypic characterization of *h*BMMSCs by flow cytometry for the expression of mesenchymal (CD90, CD73, CD105/endoglin), hematopoietic (CD34, CD45) and HLA-DR markers (black line: unstained control; red, green and purple line: marker of interest). Results are means of triplicates (±SD) of three independent experiments.

The isolated *h*BMMSCs displayed a SC phenotype, and had a comparatively high purity; practically all cells showed a positive expression of the mesenchymal markers CD73, CD90 and CD105 (>95%) and lack expression of the hematopoietic markers, CD34, CD45 and HLA-DR (<5%) (Figure 1).

### 3.2. Cell Proliferation and Attachment

**Figure 2:**
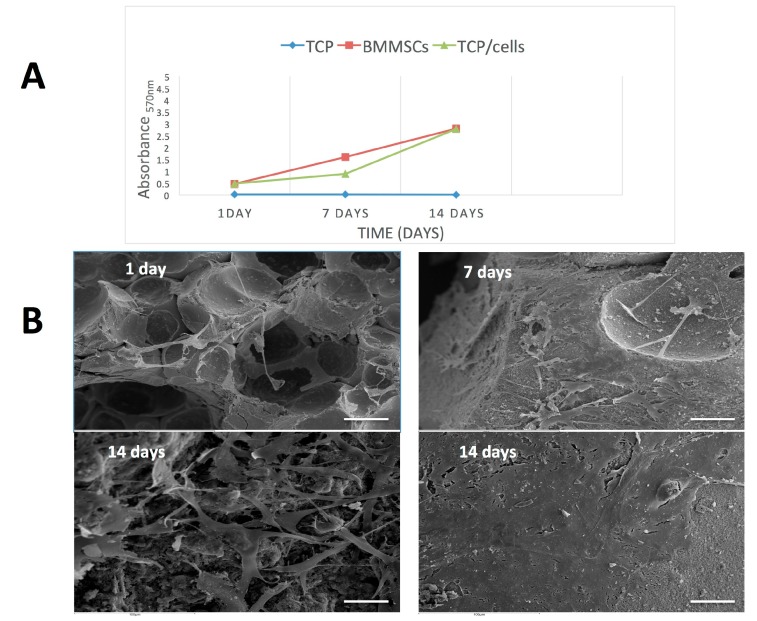
(**A**) MTT assay results of *h*BMMSCs and β-TCP/*h*BMMSCs construct. Results are expressed as relative MTT activity compared with the control. Data were shown as mean ± SD from three independent experiments; (**B**) Cellular shape and adherence of *h*BMMSCs onto β-TCP by scanning electron microscopy (SEM) 1, 7 and 14 days post-seeding on β-TCP. Scale bar: 100 μm.

Figure 2A shows the proliferation of *h*BMMSCs on β-TCP after 1, 7 and 14 days, as assessed by the MTT assay. *h*BMMSCs incubated in culture plates were monitored as positive control and cell-free scaffolds incubated under the same conditions were used as negative control. An MTT assay was performed at days 1, 7 and 14 after cell seeding into β-TCP to assess cell survival and proliferation. A significant increase in MTT reduction was seen at day 14 compared with days 1 and 7, indicating that *h*BMMSCs were able to survive and proliferate on β-TCP granules (*p* < 0.01). In addition, the PDN obtained with and without β-TCP was 2.22 ± 0.18 versus 2.09 ± 0.15, respectively.

SEM analyses revealed that small quantities of *h*BMMSCs were evenly attached to β-TCP granules after 24 h (Figure 2B). Importantly, at longer culture times (7 days) the hBMMSCs covered all the biomaterial, exhibiting a fibroblastoid morphology with several cytoplasmatic prolongations that allow the cells to anchor to β-TCP surface and establish intercellular connections. After 14 days of culture, large amounts of *h*BMMSCs adhered to the β-TCP granules, appearing as multilayered cultures. Moreover, calcified matrix deposition was detected on the surface of the cells.

### 3.3. Acute, Subchronic Toxicity Study

**Figure 3:**
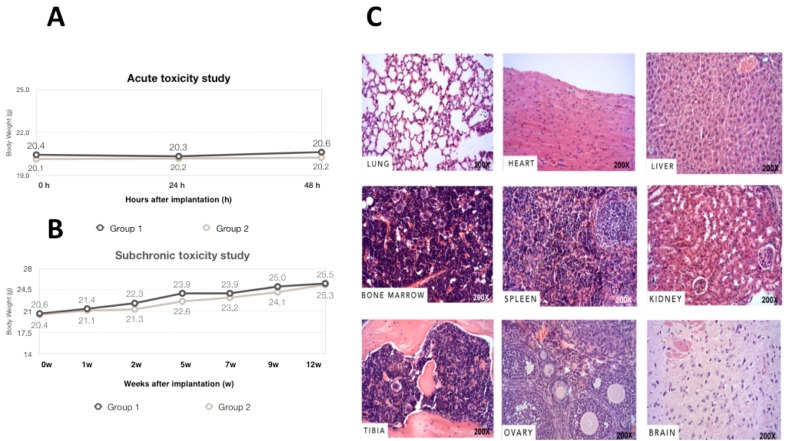
The body weight changes of the NOD/SCID mice after construct implantation for (**A**) 48 h (Acute Toxicity study) and (**B**) 12 weeks (Subchronic Toxicity study); (**C**) Histological analysis of various organs collected (lung, heart, liver, bone marrow, spleen, kidney, tibia, ovary and the brain). No structural changes or injuries were detected in theses organs.

No death or clinical signs associated with toxicity occurred during the 48-hour observation period in animals. Mice exhibited normal behavior, without surgery complications or abnormal locomotor activities. No abnormal form or color was found in the animals’ feces. Body weight changes were measured during this 2-day period. The welfare score of the 30 mice prior to and post-implantation was 0. According to Figure 3A, no statistically significant weight loss was observed between *h*BMMSCs/β-TCP group and the physiological saline control group (*p* = 0.820).

Local and subchronic toxicity of *h*BMMSCs/β-TCP constructs were assessed in a 12-week toxicity study. No mortalities or adverse clinical signs were found in both groups (Figure 3B). There was no significant difference in body weight between groups in each week. Dose-related change in mean daily food or water consumption was not observed between the negative control and the *h*BMMSCs/β-TCP groups throughout the experimental period. Additionally, no macroscopic findings were observed at necropsy, and microscopic analysis (Figure 3C) revealed no histopathological or tumor alterations in any of the paraffin-embedded tissues.

### 3.4. Biodistribution

DNA extraction and PCR analysis were performed to detect the presence of human cells in mouse tissues. The results are expressed as the number of mice (or percentage) with PCR positive for human beta-globin gene. Our results showed the presence of human cells on scaffolds during all experiment (24 h to 12 weeks post-implantation) (Table 1). However, we did not detect human cells in lung, heart, kidney, spleen, tibialis anterior muscle, brain, inguinal fat pad, bone marrow, stomach, intestine, liver, ovary, blood, skin or the knee joint.

### 3.5. In Vivo Bone Formation

**Figure 4:**
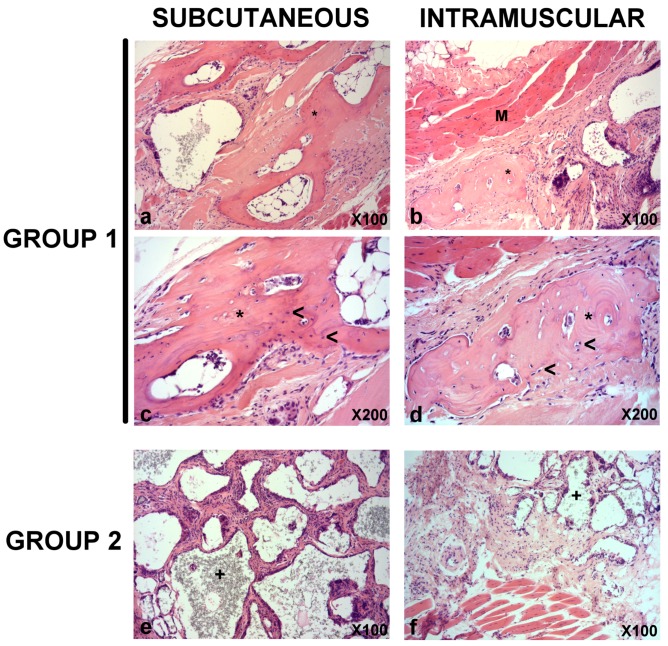
Representative images of subcutaneous and intramuscular constructs from Group 1 (**a**–**d**) and Group 2 (**e**,**f**) at week 9 after constructs implantation. While there was signs of formation of lamellar bone (asterisks) with signs of functional lacunae (presence of nucleus, head arrows) interspersed within the construct matrix in subcutaneous (**a**,**c**) and intramuscular (**b**,**d**) constructs from Group 1, in subcutaneous (**e**) and intramuscular (**f**) constructs from Group 2 there was a infiltration of connective tissue with trabecular disposition in which signs of a refringent material (+) could be identified within trabeculae. (M): Skeletal muscle. Hematoxylin and eosin (H&E) stain. Magnifications: 100 × (**a**,**b**,**e**,**f**) and 200 × (**c**,**d**).

Next, the in vivo bone formation was analyzed. Histopathological analysis revealed signs of lamellar bone formation in both the subcutaneous (from week 7) and intramuscular (from week 9) constructs of Group 1 (*h*BMMSCs/β-TCP, Figure 4). No signs of lamellar bone neoformation were observed in the subcutaneous and intramuscular constructs of Group 2 at any time.

**Figure 5:**
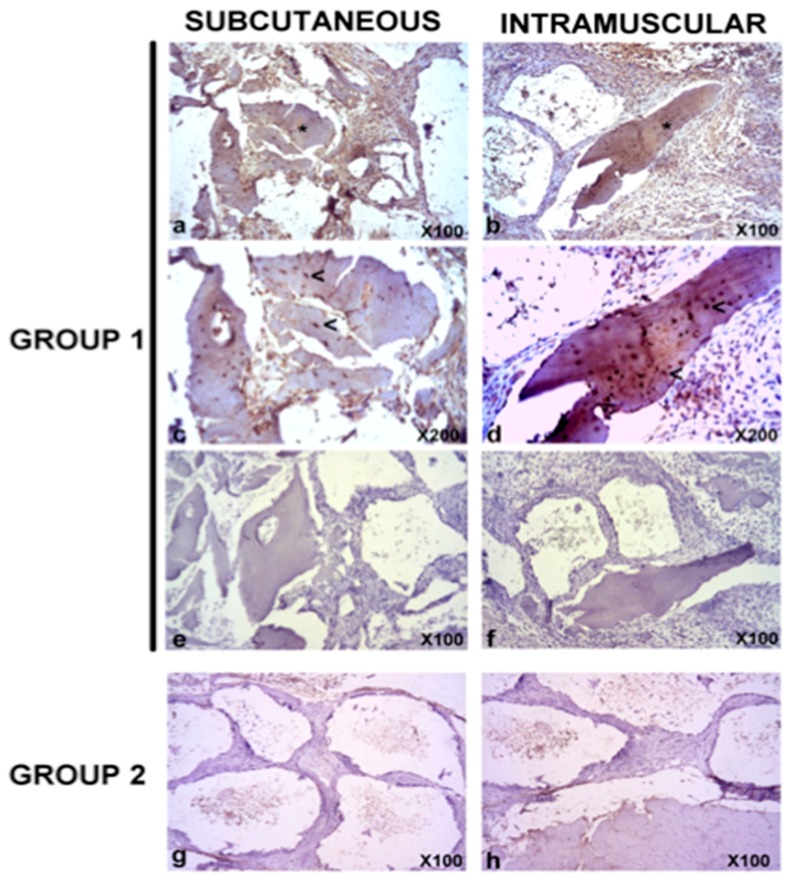
Representative images of human osteocalcin expression of subcutaneous and intramuscular constructs from Group 1 (**a**–**f**) and Group 2 (**g**,**h**) at week 9 after constructs implantation. There was positive expression of human osteocalcin in lamellar bone formations within subcutaneous or intramuscular constructs from Group 1 (**a**,**b** asterisks), particularly in functional lacunae (**c**,**d**, head arrows). Any sign of background was observed in negative controls of the same regions (**e**,**f**). On the other hand, no signs of positive immunoreaction were observed in subcutaneous and intramuscular constructs from Group 2 (**g**,**h**). ABC anti-human osteocalcin stain. Magnifications: 100 × (**a**,**b**,**e**–**h**) and 200 × (**c**,**d**).

Immunohistochemical expression of human osteocalcin was detected only in bone marrow from mice of Group 1 from week 7 onwards (Figure 5). On the other hand, no signs of positive immunoreaction were observed in subcutaneous and intramuscular constructs from Group 2.

**Figure 6:**
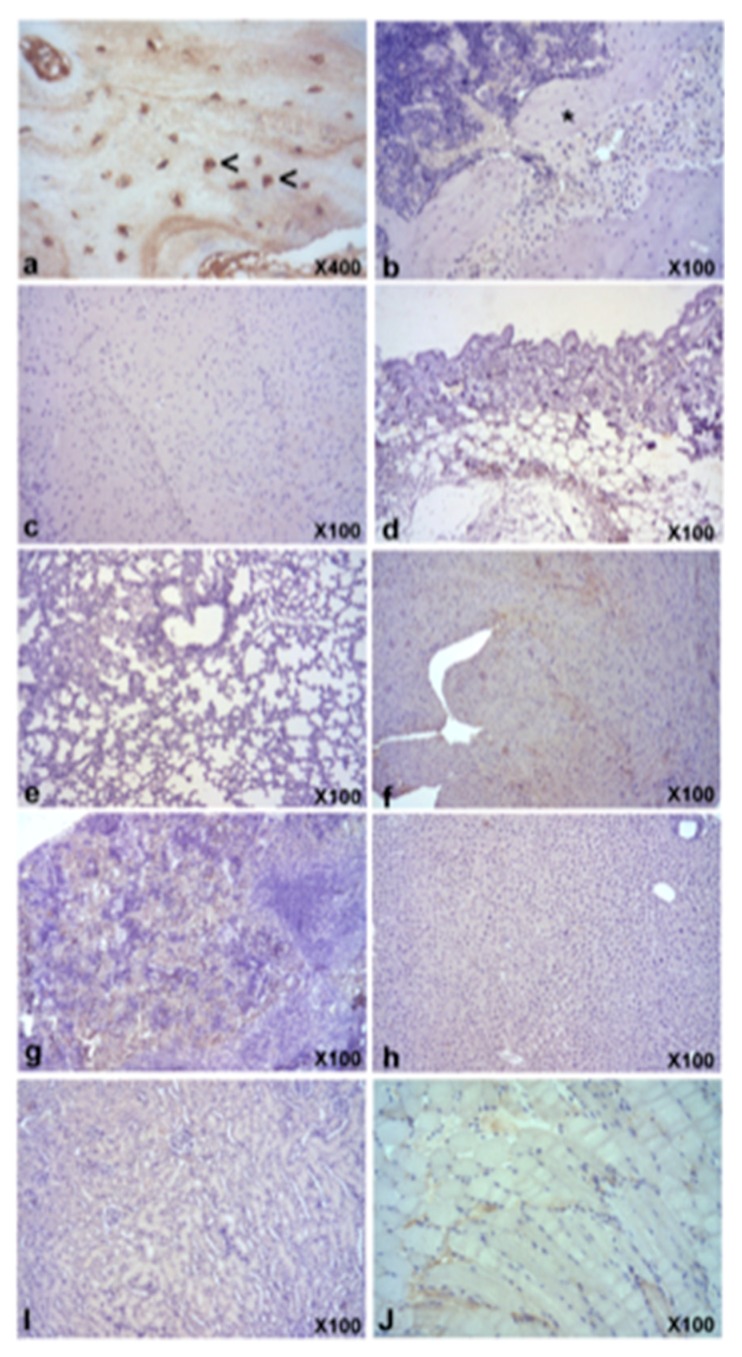
Representative images of expression of human osteocalcin in human bone (positive control, **a**); and in mouse bone (**b**); brain (**c**); skin (**d**); lung (**e**); heart (**f**); spleen (**g**); liver (**h**); kidney (**I**) and skeletal muscle (**j**). While there is a strong positive expression of osteocalcin in lacunae in human control bone (**a**, head arrows), no positive reaction was observed neither in bone (**b**), nor other tissues (**c**–**j**) in the mouse. ABC anti-human osteocalcin stain. Magnifications: 100 × (**a**,**b**,**e**–**j**).

Quantitative results (Table 2) exhibited a significant difference in the osteocalcin expression among the subcutaneous/intramuscular group (Group 1) and control group (Group 2) (*p* < 0.05). There was no significant difference between the subcutaneous and intramuscular localizations. Overall, the results indicated that the *h*BMMSCs/β-TCP group can promote the formation of calcified matrix and the osteocalcin expression compared with the control group. While there is a strong positive expression of osteocalcin in lacunae in human control bone, no positive reaction was observed either in bone or other tissues in the mouse (Figure 6).

## 4. Discussion

Preclinical studies of the products for use in new CBT need to be carried out in animal models in order to verify their biosecurity and efficacy [25]. In fact, determining the distributive fate and retention of CBT products after administration is key part of characterizing their mechanism of action and security profile [25,26]. The present study was prepared to analyze the biosafety of hBMMSCs pre-seeded into TCP scaffolds after subcutaneous/intramuscular transplantation.

We reported that (i) hBMMSCs/β-TCP constructs did not cause acute or subchronic toxicities to the mice (inspection of the health status of the operated mice and histologically analyses of several tissue samples); (ii) human cells do not migrated into tissues distant from the implantation sites (expression of human globin gene, by quantitative PCR, in several tissues); (iii) hBMMSCs/β-TCP constructs developed into bone tissue.

The limitation of this study was the animal model; immunocompetent animal model made the evaluation of the immune response of the implanted hBMMSCs under Good Laboratory Practice (GLP) conditions difficult and could be more significant by investigating the impact of SCs in larger animal models. In contrast, subcutaneous implantation is an easy and non-invasive technique, and allows performance of several test items in the same animal [27].

New materials must first manifest their biocompatibility before cells can proliferate and produce an extracellular mineralized matrix on a substrate [28]. For this purpose and to evaluate the possible cytotoxicity of the β-TCP, we investigated the viability and cell attachment of hBMMSCs cultured on β-TCP by MTT assay and SEM, respectively. A similar level of cell viability to the control was seen after 14 days of culture. Previous studies using colorimetric assays demonstrated good metabolic cell activity, cell adhesion and cell morphology promoted by β-TCP [29,30,31]. SEM is the most commonly used electron microscopy approach to analyze morphological appearance of cells seeded on certain biomaterials prior to implantation [32]. After 14 days of culture, we observed large amounts of hBMMSCs adhering to the β-TCP granules, giving the appearance of multilayered cultures. Arpornmaeklong et al. [33] showed that β-TCP stimulates the attachment and differentiation of human embryonic SCs (hESCs), especially the expression of genes related to neurogenesis (AP2a, FoxD3, HNK1, P75, Sox1, Sox10). Another recent study exhibited good morphology and cell attachment of dental pulp SCs into the β-TCP scaffolds [34].

Therapies based on SCs have shown great potential in many clinical studies. However, novel therapies using cell-based ATMPs require special safety testing strategies [27]. Thus, any additional information showing toxicity tests can help guide the design of clinical trials [35]. In our study, the local and systemic toxicity of *h*BMMSCs intramuscular and subcutaneous transplanted was monitored for 12 weeks. No mortality, morbidity or abnormal clinical symptoms were found. Moreover, no *h*BMMSC-related changes were observed in histopathological lesions. In a previous study involving mesenchymal progenitor cells derived from umbilical cord blood intravenously administered in mice, no toxicologically meaningful microscopic findings were observed in the animals [36]. Importantly we did not observe any tumors in the sacrificed animals.

Due to the cell migration after local administration, biodistribution studies are key elements for understanding the physiological or pathological behavior of the cells before clinical use [37]. Our biodistribution results did not show any *h*BMMSCs in the tested organs (lung, heart, kidney, spleen, tibialis anterior muscle, brain, inguinal pad, bone marrow, stomach, intestine, liver, ovary, blood, knee joint) 12 weeks after transplantation, suggesting that cells stay where they are placed and do not invade other tissues. These data were consistent with those of a previous study in which no hDNA was detected in such major organs as the brain, heart, lungs, kidneys, spleen or liver of animals after intramuscular administration of hMSCs [25]. In the same line, Choi et al. [38] reported that intracranially injected adipose mesenchymal SCs did not invade other tissues out of the brain in normal mice. However, after intra-articular injection, human Alu sequences were detected in several tissues and organs [39]. This suggests that the biodistribution potential of mesenchymal SCs could be influenced by the route of administration.

Regarding the efficacy of *h*BMMSCs, subcutaneous ectopic bone formation models are commonly used by CBT [40,41]. Previous reports have demonstrated that murine or human bone marrow stromal cells seeded on calcium phosphate (CaP) stimulate the bone formation implanted subcutaneously in immune-compromised mice [42]. Our results showed the presence of signs of lamellar bone formation in both subcutaneous and intramuscular constructs of group β-TCP + *h*BMMSCs from week 7 in those cases of subcutaneous implantation, and from week 9 in the intramuscular implants. While there was a strong positive expression of osteocalcin in lacunae in human control bone, a positive reaction was observed neither in bone, nor other tissues in the mouse. In this context, other authors have shown the therapeutic efficacy of BMMSCs/β-TCP in goat models of critical size bone defects [43].

## 5. Conclusions

Based on the data described in this work, it is concluded that transplantation of mesenchymal stem cell from bone marrow preseeded into β-TCP scaffolds in murine models is safe and effective. This results pave the way to perform “first in human” clinical trials to treat bone diseases in the future.

## Figures and Tables

**Table 1:** Biodistribution of *h*BMMSCs using the quantitative polymerase chain reaction (qPCR) technique, for 12 weeks. Controls: cell-free scaffolds; (+), detection of human RNAse P gene; (−), no detection of human RNAse P gene.

Tissues/Organs	24 h	1 w	2 w	5 w	7 w	9 w	12 w	Controls
Scaffold	+	+	+	+	+	+	+	−
Lung	−	−	−	−	−	−	−	−
heart	−	−	−	−	−	−	−	−
kidney	−	−	−	−	−	−	−	−
spleen	−	−	−	−	−	−	−	−
tibialis anterior muscle	−	−	−	−	−	−	−	−
brain	−	−	−	−	−	−	−	−
inguinal fat pad	−	−	−	−	−	−	−	−
bone marrow	−	−	−	−	−	−	−	−
stomach	−	−	−	−	−	−	−	−
intestine	−	−	−	−	−	−	−	−
liver	−	−	−	−	−	−	−	−
ovary	−	−	−	−	−	−	−	−
blood	−	−	−	−	−	−	−	−
skin	−	−	−	−	−	−	−	−
knee joint	−	−	−	−	−	−	−	−

**Table 2:** Frequency of human osteocalcin expression in mice after construct implantation compared with the control. ** *p* < 0.01.

Intramuscular Implant			Subcutaneous Implant		
	Yes	No		Yes	No
Group I (hBMMSCs/TCP)	*n* = 18 **	*n* = 7	Group I (hBMMSCs/TCP)	*n* = 17 **	*n* = 8
Group II (Cell free TCP)	0	*n* = 5	Group II (Cell free TCP)	0	*n* = 5
